# The Transcription Profile of Tax-3 Is More Similar to Tax-1 than Tax-2: Insights into HTLV-3 Potential Leukemogenic Properties

**DOI:** 10.1371/journal.pone.0041003

**Published:** 2012-07-20

**Authors:** Sébastien A. Chevalier, Stéphanie Durand, Arindam Dasgupta, Michael Radonovich, Andrea Cimarelli, John N. Brady, Renaud Mahieux, Cynthia A. Pise-Masison

**Affiliations:** 1 Virus Tumor Biology Section, Laboratory of Cellular Oncology, National Cancer Institute, National Institutes of Health, Bethesda, Maryland, United States of America; 2 Retroviral Oncogenesis laboratory, Ecole Normale Supérieure, INSERM U758, Lyon, France; 3 Laboratory of Primate Lentiviruses, Ecole Normale Supérieure, INSERM U758, Lyon, France; George Mason University, United States of America

## Abstract

Human T-cell Lymphotropic Viruses type 1 (HTLV-1) is the etiological agent of Adult T-cell Leukemia/Lymphoma. Although associated with lymphocytosis, HTLV-2 infection is not associated with any malignant hematological disease. Similarly, no infection-related symptom has been detected in HTLV-3-infected individuals studied so far. Differences in individual Tax transcriptional activity might account for these distinct physiopathological outcomes. Tax-1 and Tax-3 possess a PDZ binding motif in their sequence. Interestingly, this motif, which is critical for Tax-1 transforming activity, is absent from Tax-2. We used the DNA microarray technology to analyze and compare the global gene expression profiles of different T- and non T-cell types expressing Tax-1, Tax-2 or Tax-3 viral transactivators. In a T-cell line, this analysis allowed us to identify 48 genes whose expression is commonly affected by all Tax proteins and are hence characteristic of the HTLV infection, independently of the virus type. Importantly, we also identified a subset of genes (n = 70) which are specifically up-regulated by Tax-1 and Tax-3, while Tax-1 and Tax-2 shared only 1 gene and Tax-2 and Tax-3 shared 8 genes. These results demonstrate that Tax-3 and Tax-1 are closely related in terms of cellular gene deregulation. Analysis of the molecular interactions existing between those Tax-1/Tax-3 deregulated genes then allowed us to highlight biological networks of genes characteristic of HTLV-1 and HTLV-3 infection. The majority of those up-regulated genes are functionally linked in biological processes characteristic of HTLV-1-infected T-cells expressing Tax such as regulation of transcription and apoptosis, activation of the NF-κB cascade, T-cell mediated immunity and induction of cell proliferation and differentiation. In conclusion, our results demonstrate for the first time that, in T- and non T-cells types, Tax-3 is a functional analogue of Tax-1 in terms of transcriptional activation and suggest that HTLV-3 might share pathogenic features with HTLV-1 *in vivo*.

## Introduction

Among complex deltaretroviruses, Human T-cell Lymphotropic Viruses type 1, 2 and 3 (HTLV-1, -2 and -3), together with their simian counterparts STLV (Simian T-cell Lymphotropic Viruses), form the PTLV group (Primate T-cell Lymphotropic Viruses). HTLV-1 is present in endemic areas such as Southern Japan, sub-Saharan Africa, the Caribbean, South-America and Oceania, and infects ten to twenty million people worldwide. The virus is primarily transmitted through breast-feeding but can also be spread through sexual intercourse and contaminated fluids [1]. HTLV-1 is the etiological agent of Adult T-cell Leukemia/Lymphoma (ATLL), a malignant lymphoproliferation of T CD4^+^ cells [2], [3] and of HTLV-1 Associated Myelopathy/Tropical Spastic Paraparesis, a chronic neurological inflammatory disease (HAM/TSP) [4], [5], [6].

**Figure 1:**
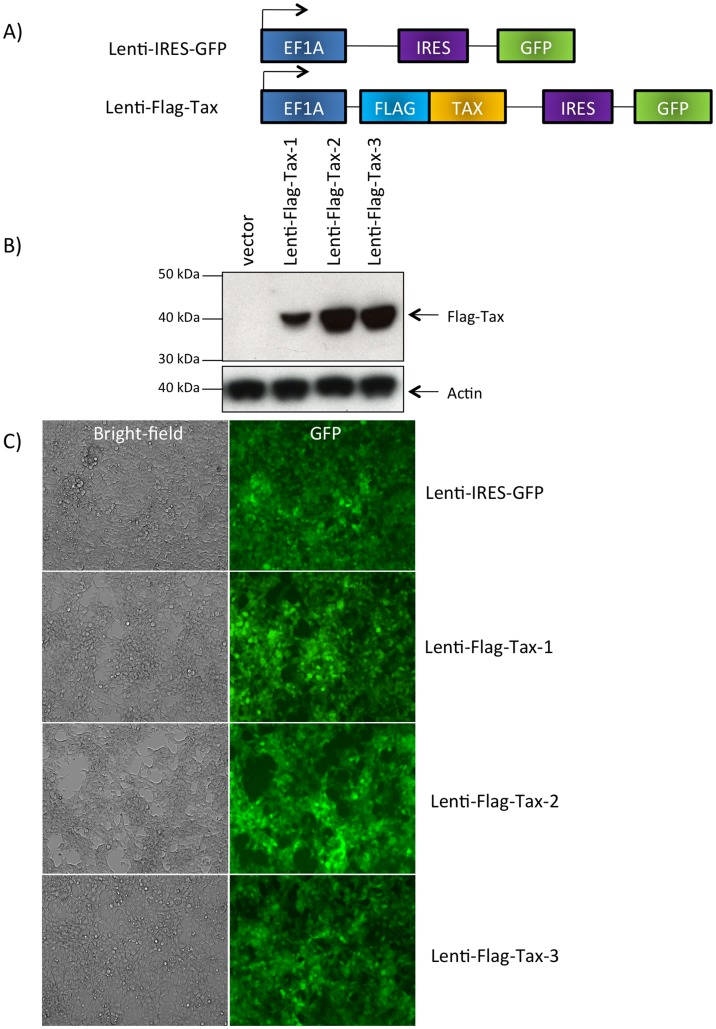
Expression of the Lenti-Flag-Tax lentiviruses. (A) Schematic representation of the Lenti-IRES-GFP and Lenti-Flag-Tax constructs. (B) Western blot analyses were performed on 70 µg of cellular extracts from MOLT4 cells transduced for 72 h by Lenti-IRES-GFP, Lenti-Flag-Tax-1, Lenti-Flag-Tax-2 or Lenti-Flag-Tax-3 lentiviruses, as indicated. Membranes were probed with anti-Flag-M2 or anti-β-actin antibody (Sigma). (C) 293 T cells were transduced by Lenti-IRES-GFP or Lenti-Flag-Tax lentiviruses for 72 h. Pictures of live cells were taken using Nikon eclipse TS 100 microscope (magnification x10).

Endemic HTLV-2 is found in Pygmies from Central Africa [7] as well as in Amerindians from North-, Central- or South-America, but also circulates among intravenous drug users in the USA, in Europe and in South Asia [4]. This virus is estimated to infect 1 to 5 million people worldwide. Importantly, although HTLV-2 infection is associated with lymphocytosis, it has not been shown to cause any malignant hematological disease so far. HTLV-2 infection is associated with cases of “HAM/TSP-like” diseases [8], [9]. Unfortunately, possible misdiagnoses and confounding factors like being an Intravenous Drug User (IDUs) and concomitant HIV-1 infection have not permitted the establishment of a clear association of HTLV-2 infection with this neurological disease [10].

**Figure 2:**
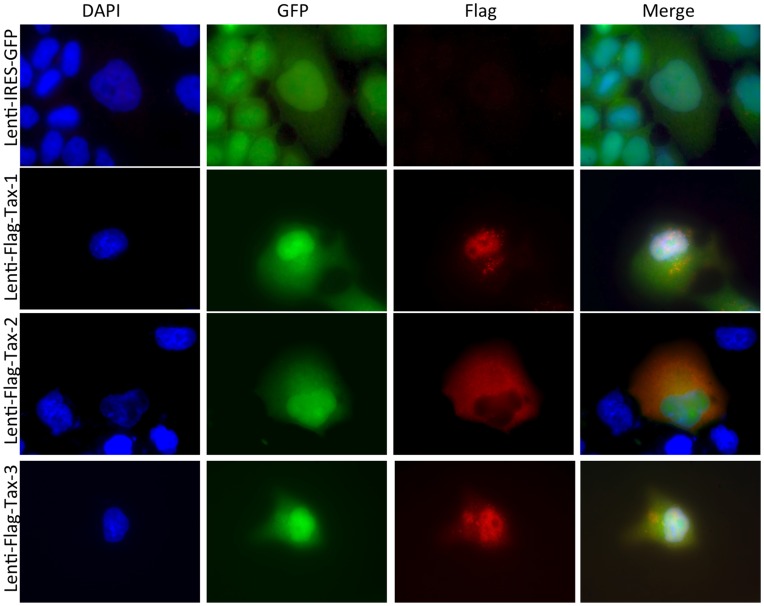
Intracellular localization of the Flag-Tax proteins. 293 T cells were transduced by Lenti-IRES-GFP or Lenti-Flag-Tax lentiviruses for 72 h. Cells were fixed on Lab-tek slides and stained with the mouse monoclonal antibody anti-Flag-M2 (Sigma) followed by a goat anti-mouse CY3-conjugated secondary antibody (Amersham Biosciences). Nucleic acids were stained with DAPI-containing mounting medium (Vectashield, Vector Laboratories). Cells were vizualized using with a Zeiss Axioplan 2 imaging microscope, X40, and the SimplePCI software (Hamamatsu).

**Figure 3:**
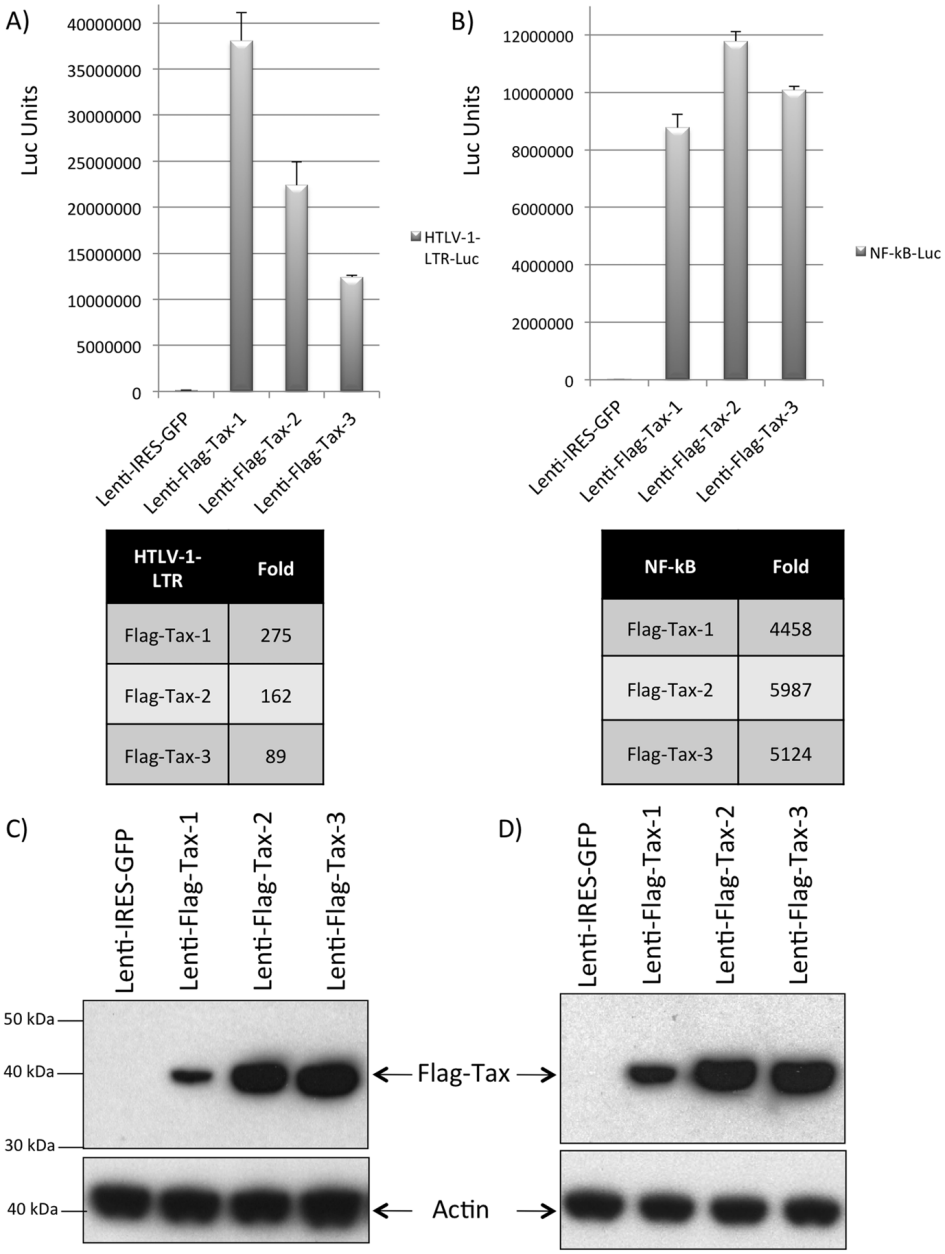
Transcriptional activity of the Flag-Tax proteins on the HTLV-1 promoter or on a synthetic NF-κB **promoter.** (A, B): 293 T cells were transduced by Lenti-Flag-Tax lentiviruses for 48 h. Cells were then transfected with (A) 500 ng of HTLV-1-LTR-Luc or (B) 500 ng of NF-κB-Luc reporter plasmid. Transfection results were normalized to Renilla activity by transfecting 10 ng of phRG-TK (Promega), and DNA quantities were adjusted with vector control. The results presented in (A) and (B) are the average of at least 3 independent experiments. Tables represent the fold-activation of the different Flag-Tax proteins over the control. (C, D) Western blot analyses were performed on 70 µg of cellular extracts from 293 T cells transduced in A and B. Membranes were probed with anti-Flag-M2 or anti-β-actin antibody (Sigma).

In 2005, two independent laboratories reported the discovery of HTLV-3 [11], [12]. Since then, two other strains of HTLV-3 were described [13], [14]. However, no pathology has been associated with the infection as yet [15].

All HTLV viruses possess an ORF encoding the viral Tax transactivator which is essential for proviral gene expression from the viral promoter. To date the majority of studies have focused on Tax-1, while a few were performed on Tax-2 and Tax-3 [4], [16], [17]. Several reports have shown that, in addition to regulating viral gene expression, Tax-1 regulates the expression and function of a number of cellular genes and proteins which control cellular proliferation and checkpoint control [18]. Indeed Tax-1 oncogenic potential was ascribed to its ability to deregulate cellular genes [19]. Tax-1, together with the anti-sense encoded product HBZ, participate in cell proliferation *in vivo* and *in vitro* [20]. Of note, HTLV-2 also encodes an antisense transcript that is expressed *in vivo*, is able to repress transcription from the 5′LTR but does not promote cell proliferation [21], [22].

**Figure 4:**
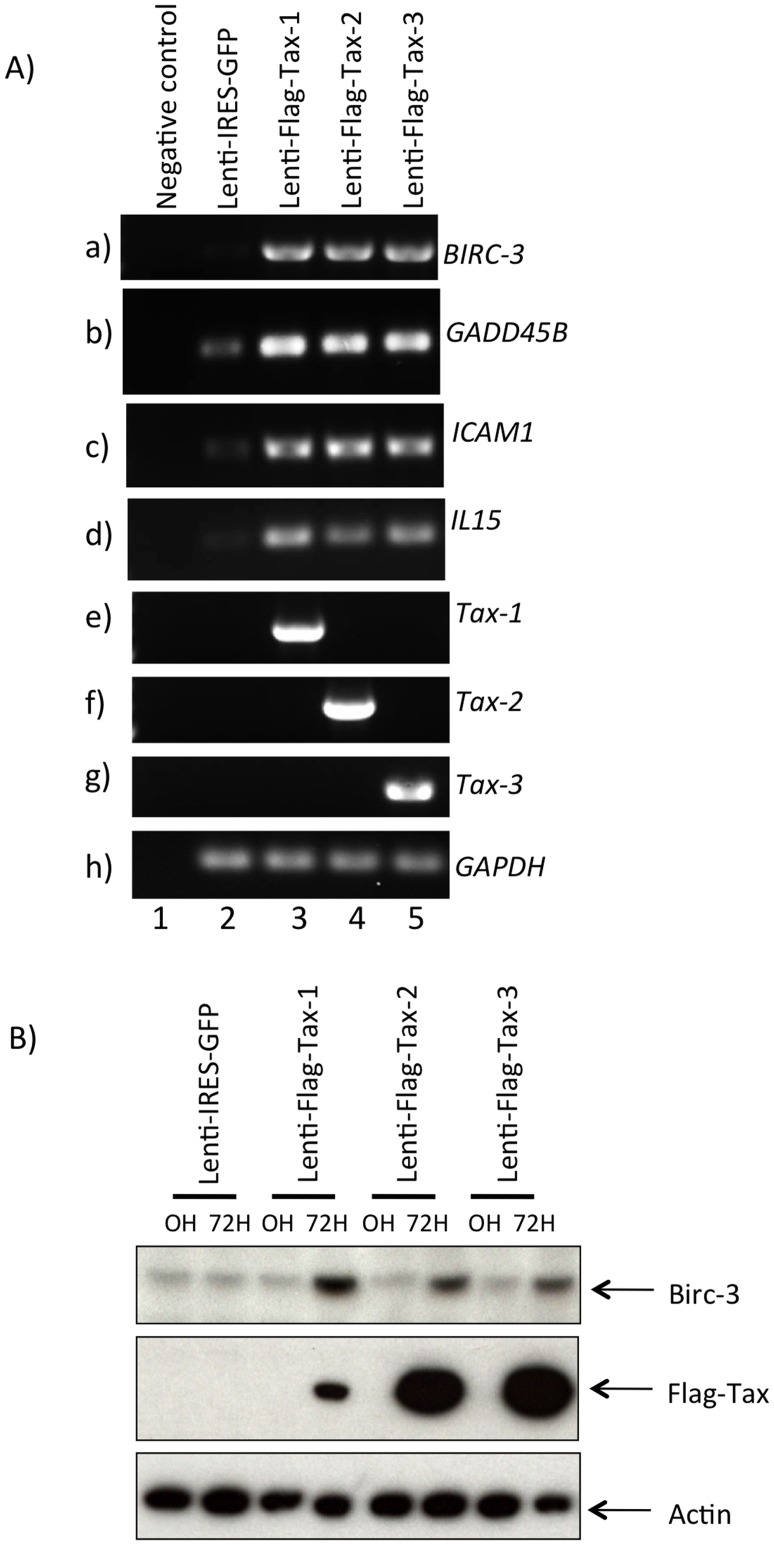
Detection of Tax and cellular genes expression by RT-PCR. (A) Total RNA was extracted from water (lane 1), or 293 T cells transduced with Lenti-IRES-GFP (lane 2), Lenti-Flag-Tax-1 (lane 3), Lenti-Flag-Tax-2 (lane 4) and Lenti-Flag-Tax-3 (lane 5). The primer sequences used for amplifying (a) *tax-1*, (b) *tax-2*, (c) *tax-3*, (d) *GAPDH*, (e) *IL-15*, (f) *GADD45B*, (g) *BIRC-3* and (h) *ICAM1* transcripts are summarized in Table S1. (B) Western blot analyses were performed on 70 µg of cellular extracts from 293 T cells transduced by Lenti-IRES-GFP, Lenti-Flag-Tax-1, Lenti-Flag-Tax-2 or Lenti-Flag-Tax-3 lentiviruses, as indicated. Membranes were probed with anti-Flag-M2, anti-β-actin or anti-BIRC-3 antibody.

Of interest, expression of Tax-1 alone is able to drive immortalization of human lymphocytes [23] and transformation of the RAT-1 cell line *in vitro* [24]. In addition, Tax-1 expression can induce an ATL-like malignancy in transgenic mice [25].

**Figure 5:**
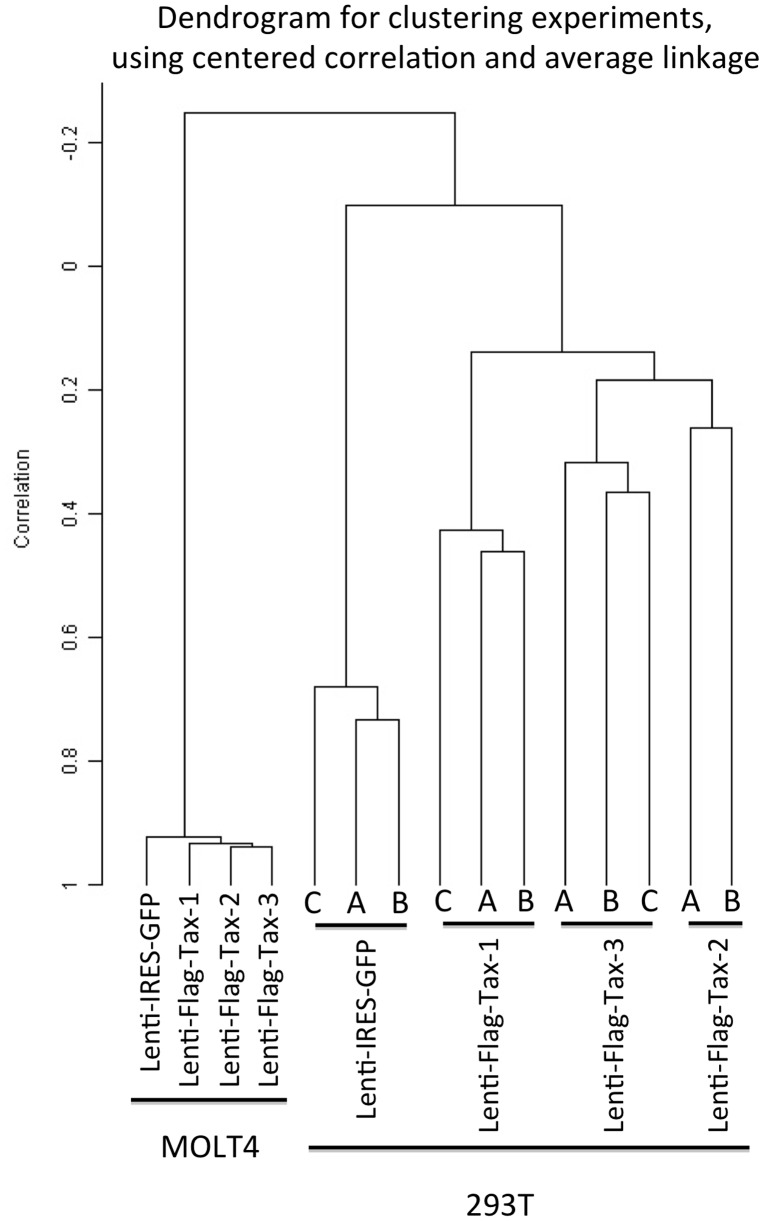
Unsupervised hierarchical clustering of Lenti-Flag-Tax-expressing 293 T and MOLT4 samples using centered correlation and average linkage. Using the BRB Array tools software (NIH, Rockville), the clustering of the different samples via the gene expression pattern in Tax-transduced MOLT4 and 293 T cells was determined. For each Lenti-IRES-GFP or Lenti-Flag-Tax construct, the dendrogram represents triplicates (A, B and C) in the transduced 293 T cells and a single experiment in the transduced MOLT4 cells. Of note, because of the quality of the sample, the third replicate of the Lenti-Flag-Tax-2 sample in the 293 T cells was excluded from the analysis. The class prediction analysis reveals that the representation of the samples can be predicted with 75% confidence based on the expression pattern of 13407 genes (p = 0,001).

Although Tax-2 shares 78% identity at the amino acid level with Tax-1, it is less efficient than Tax-1 in immortalization and transformation *in vitro* [26]. The major differences that have been described between Tax-1 and Tax-2 include: distinct intracellular localization [27], [28], [29], [30], induction of micronuclei by Tax-1 [31], induction of human CD34^+^ cells maturation *in vitro* by Tax-1 [32], strong inhibition of p53 transcriptional activity by Tax-1 but not Tax2 [33], presence of a PDZ binding motif (PBM) in the carboxyl terminal part of Tax-1 [34] as well as presence of another domain in the 225–232 Tax-1 sequence implicated in its transforming activity [35]. The PBM domain, absent from the Tax-2 protein, is critical for Tax-1 ability to transform RAT-1 fibroblast cells and deleting this domain from Tax-1 decrease its transforming potential while adding it to Tax-2 promotes RAT-1 transformation [36]. Moreover, the presence of the PBM in Tax-1 induces proliferation of human PBMCs (peripheral blood mononuclear cells) *in vitro*. In infected rabbits, a deletion of this domain in the context of an HTLV-1 molecular clone inhibits the viral persistence and the virus is eliminated within a few weeks post-infection [37]. Hence, this domain plays a key role in HTLV-1 induced cell proliferation and facilitates viral persistence *in vivo*. Finally, Tax-1 but not Tax-2 can activate both canonical and non-canonical NF-κB pathways while Tax-2 activates only the former [38]. However, an extensive comparison between Tax-1 and Tax-2 gene regulation has not been performed.

**Figure 6:**
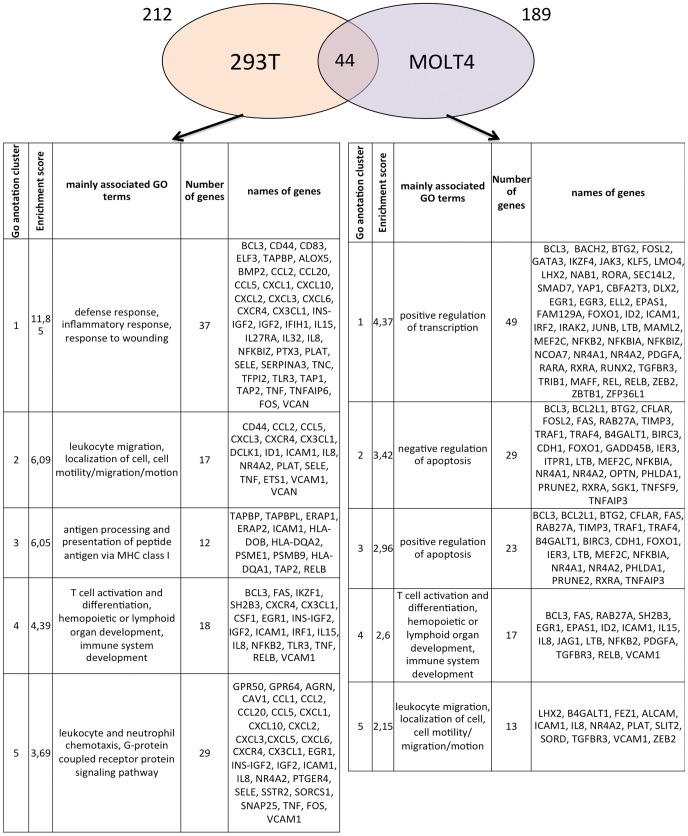
Representation of cellular genes deregulated following Tax-3 expression in 293 T and MOLT4 cells. Venn diagram representation performed on 401 cellular genes up-regulated by Tax-3 expression in 293 T and MOLT4 cells (cut-off: 3-fold over the control). For each subpopulation of genes specifically or commonly deregulated by Tax-3 expression, we performed a Gene ontology (GO) analysis using DAVID Bioinformatics web-tool (Database for Annotation, Visualization and Integrated Discovery). The first 5 clusters implicated in biological processes and possessing the highest enrichment scores were selected.

Interestingly, the first study characterizing the HTLV-3 Tax-3 viral transactivator, suggested that the protein shared sequence and function similarities with Tax-1 [16]. First, at the amino acid level, Tax-3 displays stronger similarities with Tax-1 (74%) than with Tax-2 (70%). Second, Tax-3 also possesses a PBM. This suggests that Tax-3 and Tax-1 may have similar immortalizing properties [16], [17]. In transiently transfected cells, Tax-3 displays a nuclear localization similar to that of Tax-1 [16]. Like Tax-1 and Tax-2, Tax-3 has the ability to activate the transcription from the HTLV-3 promoter via CREB/ATF but also to activate the NF-κB signaling pathway. Interestingly, like Tax-1, Tax-3 is able to transrepress the c-Myb promoter via the activation of the NF-κB pathway [16], [39].

**Figure 7:**
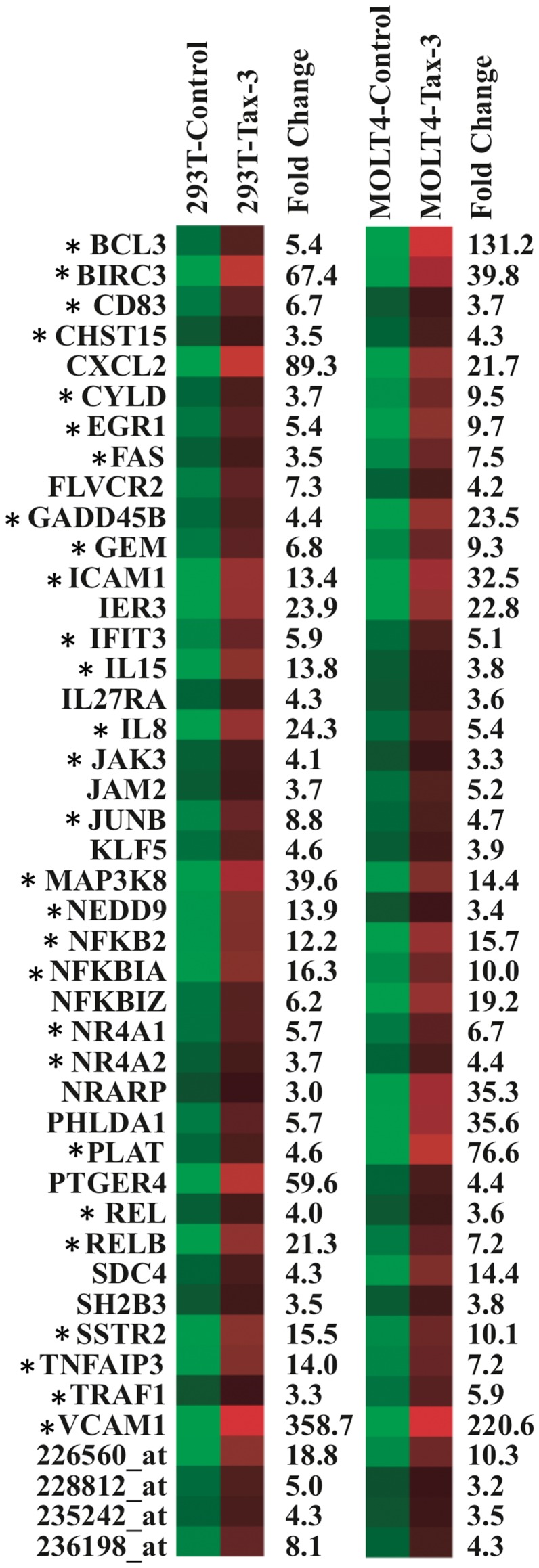
Heat Map analysis of cellular genes deregulated by Tax-3 in 293 T and MOLT4 cells. Representation of the 44 cellular genes deregulated both in 293 T and MOLT4 cells following Tax-3 expression using Heat Map analysis (log transformation and mean centered data performed in Cluster and TreeView softwares). The mean fold change expression is indicated on the right of each graphic. *Genes were already reported in HTLV literature. CHST15 is an alias of GALNAC4S-6ST.

DNA microarray technology has previously allowed the determination of gene expression profiles in HTLV-1-infected or Tax-1-expressing cells [40], [41], [42], [43], [44], [45], [46], [47], [48], [49]. So far however, GeneChip® analysis has not been performed on HTLV-2-infected samples or cell lines, or on Tax-2 or Tax-3-expressing cells (of note an HTLV-3 cell line is not available). We report here the use of this approach for the analysis of gene expression profiles of T- and non T-cells expressing Tax-1, Tax-2 or Tax-3 viral transactivators. This analysis allowed us (i) to identify a significant number of genes whose expression is commonly affected by all Tax proteins and hence characteristic of the HTLV infection, independent of the virus type; (ii) to identify a subset of genes which are specifically up-regulated by Tax-1 and Tax-3 and (iii) to demonstrate that Tax-3 and Tax-1 are closely related in terms of molecular signature on gene expression profiles.

## Results

### Lentiviral Vectors Design and Validation of Tax Expression

Since T-cells are the primary targets of HTLV infection *in vivo* and transfection of T -cells is inefficient, we introduced Tax-1, -2 or -3 sequences into the multi-cloning site of pSDM101 lentiviral vector (Dasgupta unpublished data). This vector contains the “medium” expression promoter EF1A and an IRES-GFP allowing discrimination of transduced versus non-transduced cells. Because an antibody able to detect all three Tax proteins is not available, an N-terminal Flag tag was added to the Tax sequence (Figure 1A). A T-cell line, MOLT4, and a non T-cell line 293 T, were selected to identify subset of genes deregulated independently of the cell type selected.

**Figure 8:**
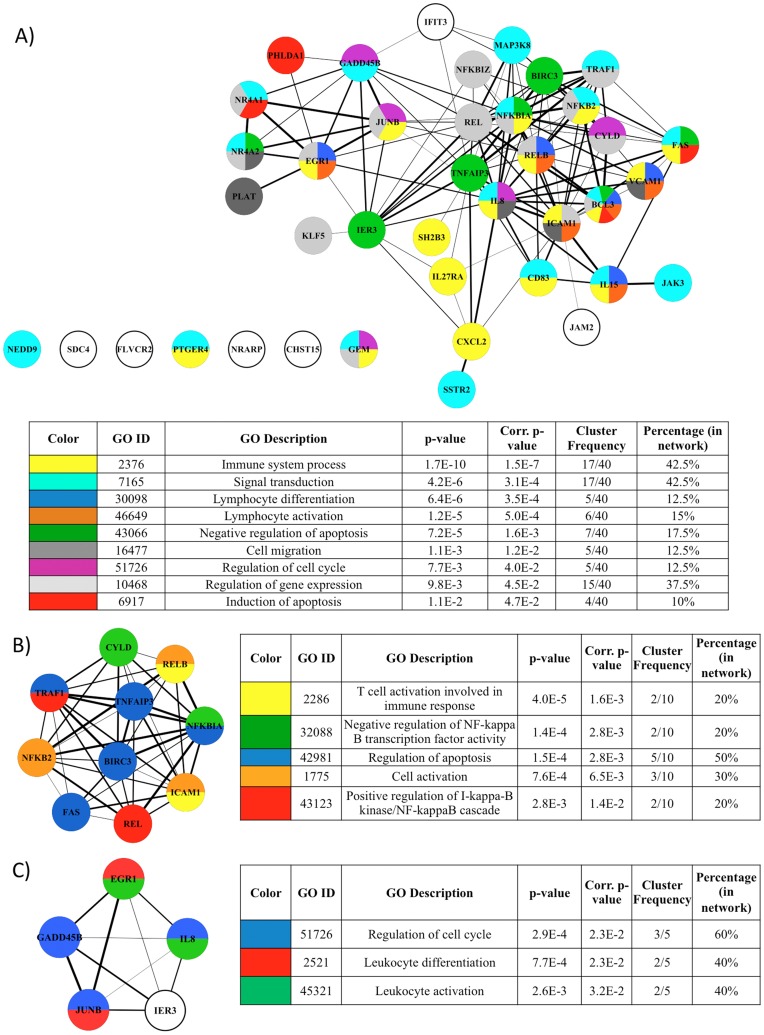
Functional analysis of cellular genes deregulated following Tax-3 expression in 293 T and MOLT4 cells. (A) Schematic representation of the 44 cellular genes implicated in molecular interactions, using the STRING software. Width of the lines reflects the score of molecular interaction and the circles are colored according to the GO Biological Process association. The color legend is indicated in the table below the network. Each color represents the main GO terms associated with genes composing the network, identified by BINGO analysis (Hypergeometric test and Benjamini & Hochberg False Discovery Rate (FDR) correction; significance level <0.05). (B, C) Sub-networks correspond to two densely connected regions of the main molecular interaction network identified by MCODE plugin. Each sub-network was re-analyzed for GO enrichment and results are indicated in the tables next to each sub-network.

In transduced MOLT4 cells (data not shown) or in 293 T (Figure 1B) cells, Flag-Tax proteins were detected by western blot at the expected molecular weight. The levels of Tax were similar but not identical. The level of Tax-1 protein was reproducibly lower than that of the two other proteins, but all Tax proteins were transcriptionally active (see below). As a control, actin western blot also demonstrated that the protein amounts loaded onto the gel were identical. The fact that despite being expressed from the same vector the different Tax proteins have different expression levels is not without precedent. Indeed, it has been previously shown that, in 293 T cells, the HTLV-2 p28 protein was expressed 25 to 30 fold higher than the HTLV-1 p30 protein. This difference was not related to differences in transfection efficiency [50]. In our case, microscopic analyses performed in 293 T (Figure 1C) and MOLT4 (data not shown) demonstrated that under those experimental conditions, more than 95% of 293 T cells were GFP positive regardless of the Tax constructs.

**Figure 9:**
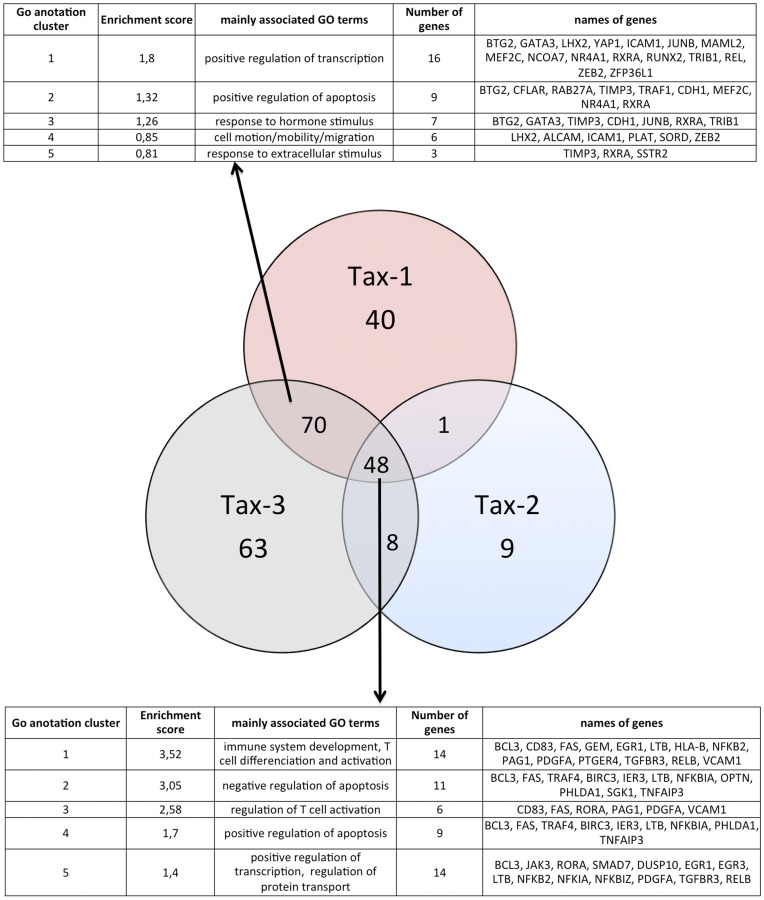
Representation of genes deregulated by the Tax-1, -2, -3 proteins in MOLT4 cells. Venn diagram representation performed on 239 cellular genes up-regulated by Tax expression in the MOLT4 cells (Cut-off: 3-fold above the control). Gene ontology (GO) analysis using DAVID Bioinformatics web-tool was performed on genes commonly deregulated following Tax1, -2 and -3 expression or specifically deregulated by Tax1 and Tax-3 expression in the MOLT4 cells. The first five clusters implicated in biological processes and possessing the highest enrichment scores were selected.

**Figure 10:**
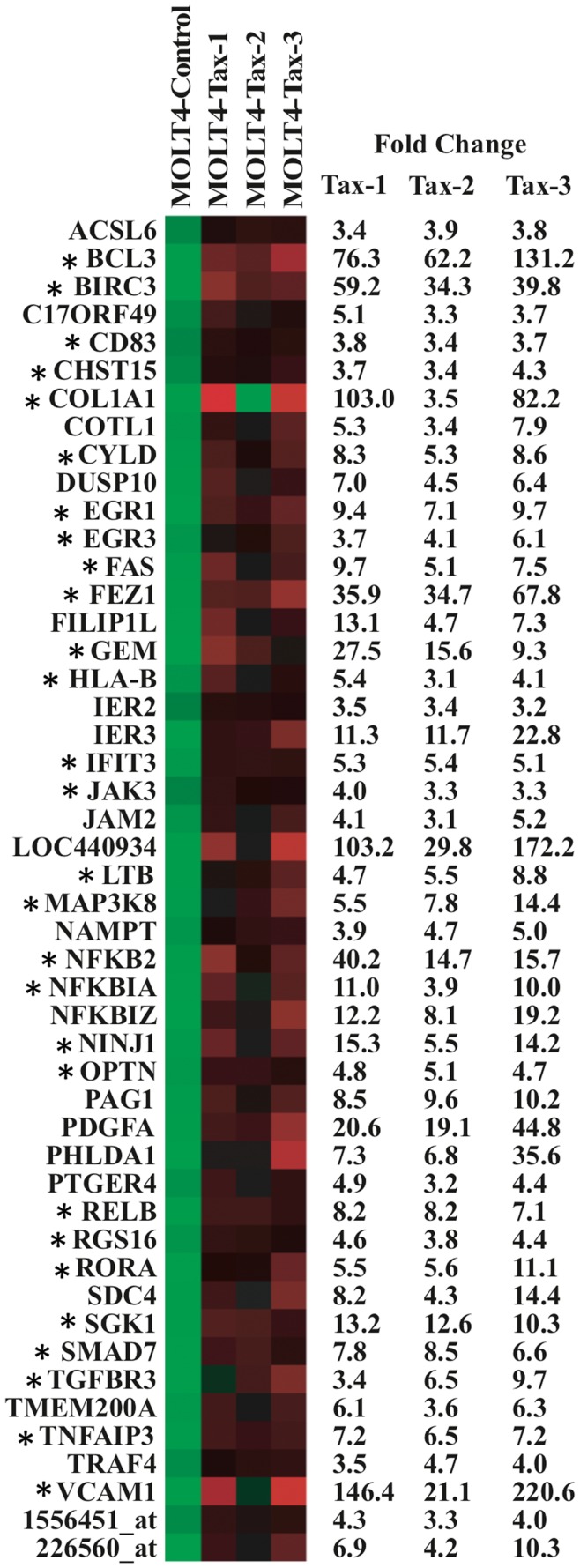
Heat Map analysis of cellular genes deregulated by the Tax-1, -2, -3 proteins in MOLT4 cells. Representation of the 48 cellular genes deregulated in MOLT4 cells following Tax proteins expression using Heat Map analysis (log transformation and mean centered data performed in Cluster and TreeView softwares). The mean fold change expression is indicated on the right of each graphic. *Genes were already reported in HTLV literature. CHST15 is an alias of GALNAC4S-6ST.

Time course experiments showed that the highest expression of these proteins occurred at 72 h post-transduction (data not shown). Hence, the following experiments and analysis were conducted 72 h post-transduction. In comparison, *Ng et al.* performed microarray experiments with the JPX-9 T-cell line between 9 and 25 h after metal-induced Tax-expression [48]. Time course experiments also showed that Tax was still expressed 4 weeks post-transduction (data not shown).

**Figure 11:**
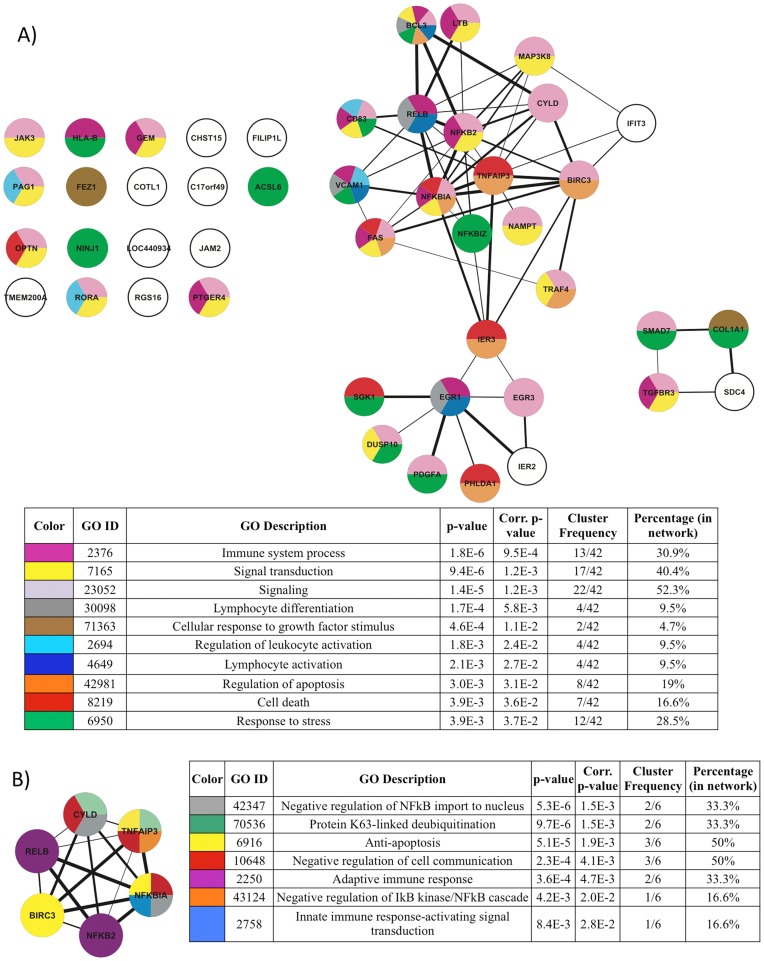
Functional analysis of genes deregulated following Tax-1, -2, -3 expression in MOLT4 cells. (A) Schematic representation of the 48 cellular genes implicated in molecular interactions, using the STRING software. Width of the lines reflects the score of molecular interaction and the circles are colored according to the GO Biological Process association. The color legend is indicated in the table below the network. Each color represents the main GO terms associated with genes composing the network, identified by BINGO analysis (Hypergeometric test and Benjamini & Hochberg False Discovery Rate (FDR) correction; significance level <0.05). (B) Sub-networks correspond to 2 densely connected regions of the main molecular interaction network identified by MCODE plugin. Each sub-network was re-analyzed for GO enrichment and results are indicated in the tables next to each sub-network.

### Characterization of Tax in Transduced Cells

To verify that Flag-Tax protein localization was similar to that of previous publications, we performed imaging of 293 T cells transduced with the different Flag-Tax lentiviral particles. As expected, these proteins exhibited an intracellular pattern characteristic of the Tax proteins *i.e.* Tax-1 and Tax-3 were localized mainly in the nucleus but also in the cytoplasm, whereas Tax-2 displayed mainly a cytoplasmic distribution (Figure 2, see Flag panel) [51], [52], [53], [54], [55]. Together these data demonstrate that the Flag tag does not alter Tax intracellular localization. Next we tested the ability of the Flag-Tax proteins to activate transcription from the HTLV-1-LTR and from a NF-κB-dependent promoter in transduced cells. Tax proteins displayed a transcriptional activity between 89 to 275 fold over the control on the HTLV-1-LTR (Figure 3A). Of note, although Tax-1 had a lower expression (Figures 3C and D), it displayed the highest transcriptional activity on this promoter. The three proteins also showed an activation of over 4000 fold on the NF-κB-responding promoter (Figure 3B). Hence, these results demonstrate that these proteins are well expressed, localized as their untagged counterparts, and transcriptionally active.

**Figure 12:**
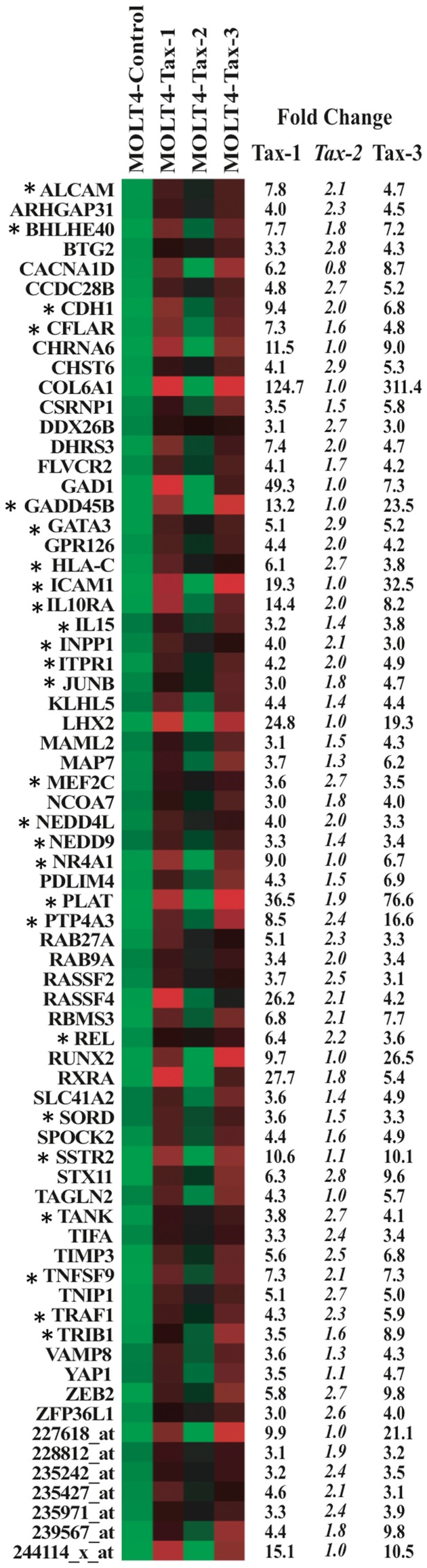
Heat Map analysis of 70 cellular genes specifically deregulated in Tax-1 and Tax-3 transduced cells. Representation of the 70 cellular genes specifically deregulated in MOLT4 cells following Tax1 and Tax-3 proteins expression using Heat Map analysis (log transformation and mean centered data performed in Cluster and TreeView softwares). The mean fold change expression is indicated on the right of each graphic. Tax-2 values were added as control. *Genes were already reported in HTLV literature. CSRNP1, BHLHE40 and CDGAP are aliases of AXUD1, BHLHB2 and ARHGAP31, respectively.

**Figure 13:**
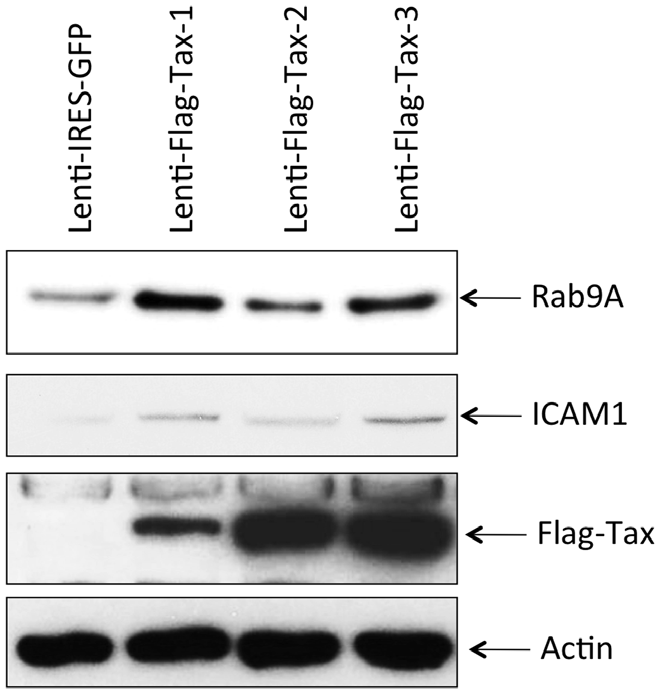
Validation at the protein level of genes deregulated in Tax-1 and Tax-3 transduced cells. Western blot analyses were performed on 70 µg of cellular extracts from MOLT4 cells transduced by Lenti-IRES-GFP, Lenti-Flag-Tax-1, Lenti-Flag-Tax-2 or Lenti-Flag-Tax-3 lentiviruses, as indicated. Membranes were probed with anti-RAB9A, anti-ICAM1, anti-Flag-M2 or anti-β-actin antibody.

### Microarray Experiments and Validation of the Gene Expression Profiles

After confirming the functionality of our Tax lentiviral constructs, we transduced MOLT4 or 293 T cells to compare transcriptional profiles in different cell types. Seventy-two hours post transduction, total RNA was collected for GeneChip® hybridation and RT-PCR analysis. Proteins were also extracted for western blot validation.

**Figure 14:**
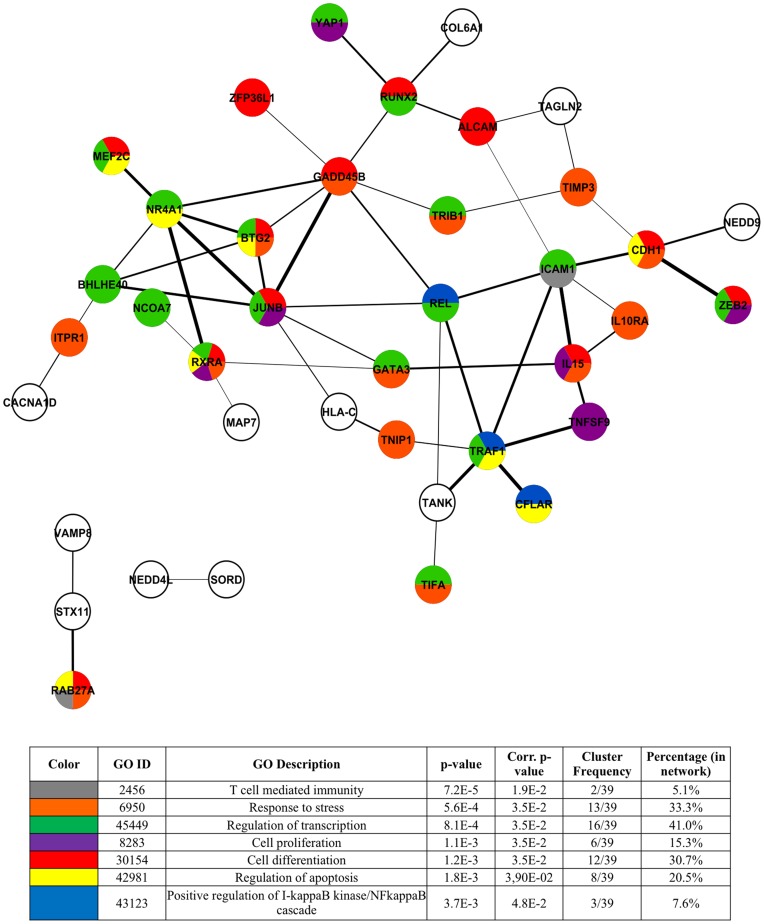
Functional analysis of 70 cellular genes deregulated only in Tax-1 and Tax-3 transduced cells. Schematic representation of the 70 cellular genes implicated in molecular interactions, using the STRING software. Width of the lines reflects the score of molecular interaction and the circles are colored according to the GO Biological Process association. The color legend is indicated in the table below the network. Each color represents the main GO terms associated with genes composing the network, identified by BINGO analysis (Hypergeometric test and Benjamini & Hochberg False Discovery Rate (FDR) correction; significance level <0.05).

The identification of up-regulated genes following Tax transduction in 293 T or MOLT4 cells was performed by comparing Tax-1, -2 or -3-transduced samples to control samples (IRES-GFP-transduced). A cut-off of 3-fold change over the control was applied. In 293 T cells, transduction of Tax-1, -2 or -3 induced the up-regulation of 268, 187 or 212 genes, respectively, corresponding to 318 unique deregulated genes (Table S2 and Figure S1). In MOLT4 cells, transduction of Tax-1, -2 or -3 increased the expression level of 159, 66 or 189 genes, respectively, corresponding to 239 unique deregulated genes (see below and Table S2). Although Tax is a viral and cellular transactivator, we also analyzed genes down-regulated in transduced-293 T and MOLT4 cells and compared them to the control. Eighty-eight, 28 and 38 genes were down-regulated (i.e. decrease of 4 fold or more) following Tax-1, -2 or -3 expression in 293 T cells respectively, while fewer (29, 15 and 42 genes for Tax-1, -2 or -3 respectively, Table S3) were down-regulated in MOLT4 cells). We focused principally on genes up-regulated following Tax transduction in subsequent analyses.

To validate the GeneChip® analysis, we performed a series of RT-PCR on a set of arbitrarily selected genes (*BIRC-3, GADD45B, ICAM-1* and *IL15)* whose expression was modulated in the microarray experiments. Levels of mRNA of all selected genes were increased in Tax-transduced cells (Figure 4A panels a, b, c and d, compare lanes 3, 4 and 5 to lane 2). As controls, *tax* and *GAPDH* RT-PCRs were also performed (Figure 4A panels e, f, g and h). We then assessed the protein expression of one of these genes (*BIRC-3*). We observed that BIRC-3 is overexpressed in Tax transduced cells but not in the control cells (Figure 4B).

Altogether, these results validate our study design and demonstrate a strong correlation between expression levels measured by hybridization on microarray and by RT-PCR. In the case of BIRC-3, alteration at the mRNA level can be extrapolated to an alteration at the protein level.

### Hierarchical Clustering Analysis Shows a Strong Linkage Among Tax-lentivirus-Transduced Cells

Unsupervised clustering analysis of samples based on the entire gene expression data sets showed a complete separation into two populations corresponding to (i) transduced MOLT4 and (ii) transduced 293 T samples (Figure 5). This suggests that individual cell types express distinct expression profiles following Tax expression. Within these 2 populations, control (Lenti-IRES-GFP) samples were distinct from Tax-transduced samples. In the case of 293 T samples that include several replicates, each group of Lenti-Flag-Tax was clearly separated, indicating differences within the gene expression profiles associated with transduction by Tax-1, -2 or -3. Furthermore, clear association of samples from a given group of replicates showed a very similar molecular profile and hence demonstrated the high reproducibility of our experiments.

Using the BRB Array Tools program, we performed a class prediction analysis with different samples obtained from Tax-transduced 293 T and MOLT4 cells. The class prediction analysis is a supervised learning method in which the algorithm predicts the class/phenotype/parameter of a sample, identifies genes that discriminate well among classes and identifies samples that could be potential outliers. By 1-nearest neighbor or 3-nearest neighbor, the representation of the samples can be predicted with 75% confidence based on the expression pattern of 13407 genes (p = 0,001). Therefore, the clustering of our different microarray experiments for a given cell line reveals a very reproducible and robust correlation among the clustering of the Lenti-Flag-Tax samples and the controls.

### Functional Analysis of Tax-3 Deregulated Genes in MOLT4 and 293 T Cells

We then focused our analysis on genes that were up-regulated in Tax-3 transduced cells (Figure 6). In MOLT4 cells, 189 genes were up-regulated, while 212 genes were up-regulated in 293 T transduced cells. To understand the biological significance of these changes at mRNA expression levels, a functional analysis was performed using the DAVID web-tool by evaluation of Gene Ontology (GO) terms enrichment (focus only Biological Process, BP, terms). Due to the high redundancy of GO terms, functional annotation clustering was done. This allowed us to cluster functionally similar terms associated with each gene list. The first more representative five clusters obtained for each subpopulation of deregulated genes with the highest enrichment score are represented in the tables below the Venn diagram (Figure 6). Despite low similarity between these two lists of genes, the functional groups of mainly associated GO terms were similar. In both cell lines, up-regulated genes were implicated in T-cell activation and differentiation as well as in leukocyte migration/mobility/motion. In addition, genes involved in antigen processing and presentation of peptide antigen via MHC class I as well as leukocyte and neutrophil chemotaxis and inflammatory response were also up-regulated in 293 T cells. On the other hand, genes involved in positive regulation of transcription and regulation of apoptosis were up-regulated in MOLT 4 cells (Figure 6).

In order to establish a gene expression profile for HTLV-3, we then compared the list of genes deregulated in MOLT4 and 293 T transduced cells. Evaluation on these two molecular profiles by Venn diagram shows that only 44 genes were commonly deregulated in both cell types (Figure 6 and Figure 7 for a list of those genes). Using Heat Map analysis, we determined that these 44 genes exhibited similarly high expression levels in both cell lines (Figure 7). Green squares indicate the basal expression level for a given gene in the control sample. Compared to the control, the lowest to the highest level of expression of each gene is represented from dark red to light-red cube and the mean fold change expression is indicated for each gene. Genes highly expressed in both cell lines, like VCAM1 which is implicated in the formation of syncitia during HTLV infection [56], or the anti-apoptotic protein BIRC3/HIAP-1/CIAP-2 that prevents the death of naturally infected HTLV-1 CD8^+^ are easily visualized with this representation [57].

Interestingly, among these 44 genes, 28 have also been previously described in the HTLV-1 literature (noted with an asterisk): *BCL3, BIRC3, CD83, CYLD, EGR1, FAS, GADD45B, GEM, ICAM1, IFIT3, IL15, IL8, JAK3, JUNB, MAP3K8, NEDD9, NFKB2, NFKBIA, NR4A1, NR4A2, REL, RELB, SSTR2, TNFAIP3, TRAF1* and *VCAM1*. However, 16 other genes have never been linked to HTLV infection: *CHST15, CXCL2* (gene encoding the CXCL2 chemokine), signal transduction genes (*CYLD, IL27RA, NFKBIZ* and *SH2B3*), *FLVCR2, IER3, JAM2* (encoding the junctional adhesion molecule 2), *KLF5* (encoding a transcription factor), *NRARP, PHLDA1* (gene implicated in regulation of apoptosis), *PLAT* (gene implicated in cell migration), *PTGER4* (encoding prostaglandin E receptor 4), *SDC* (encoding cell surface proteoglycan), and 4 Affymetrix probes that are non-associated to a functional known gene (Figure 7). Further analysis of these 16 newly identified genes will be required to determine their role in HTLV-3 infection.

Analysis of protein-protein interactions existing between each member of this selected set of genes retrieved from STRING database showed that the majority of the genes (33) can be associated in a biological network (Figure 8A). The genes composing the network were functionally linked in a series of biological processes such as immune system processes, signal transduction, lymphocyte activation and differentiation, regulation of apoptosis, cell cycle and transcription; and cell migration. Moreover, from the main molecular interaction network, two densely connected sub-networks clearly emerged (Figure 8B and C). The larger one encompasses 10 genes (*FAS, REL, NFKB2, BIRC3, ICAM1, TRAF1, TNFAIP3, NFKBIA, CYLD* and *RELB*), which are involved in regulation of apoptosis, NF-κB activation, T-cell activation and immune response (Figure 8B). The other sub-network contains 5 genes (*EGR1, IL8, IER3, GADD45B* and *JUNB*) related to biological processes such as regulation of cell cycle, activation and differentiation of lymphocyte (Figure 8C).

The functional analysis of the up-regulated genes in Tax-3 transduced cells allowed us to highlight networks of genes characteristic of HTLV-3 infection. Importantly, half of those genes were already reported in the case of HTLV-1 infection whereas, the roles of the other half of the genes identified are unknown with regards to HTLV pathogenesis.

### Functional Analysis of Genes Commonly Deregulated in MOLT4 Expressing Tax-1, Tax-2 and Tax-3

To establish a common profile of HTLV infection, we then compared gene expression profiles of MOLT4 cells transduced respectively by Tax-1, Tax-2 or Tax-3 proteins. Using Venn diagrams, we represented the distribution of the 239 up-regulated genes following Tax expression (Figure 9). Interestingly, the expression of more than 20% of the genes (i.e. 48 genes) was similarly increased by the three Tax proteins. These genes are: oncogenes/mitogens (*BCL3, MAP3K8, PDGFA, RELB, SMAD7, TGFBR3*), apoptotic/anti-apoptotic genes (*BIRC3, FAS, FILIP1L, IER3, PHLDA1, SGK1*), *CD83* gene, tumor suppressor genes (*EGR1, EGR3*), signal transduction genes (*CYLD, DUSP10*, *GEM, JAK3, NFKB2, NFKBIA, NFKBIZ, OPTN, PAG1, PTGER4, RGS16, SDC4, TNFAIP3, TRAF4*), immune response genes (*HLA-B*, *IFIT3, LTB*), cell adhesion genes (*COL1A1, JAM2, VCAM1*), genes implicated in reorganization of the cytoskeleton (*COTL1, FEZ1*) (Figure 10). Although 28 of those genes were already associated with HTLV-1 infection (as indicated by an asterisk), 20 were not yet described: *ACSL6, C170RF49, COTL1, DUSP10, FILIP1L, IER2, IER3, JAM2, LOC440934, NAMPT, NFKBIZ, PAG1, PDGFA, PHLDA1, PTGER4, SDC4, TMEM200A, TRAF4* and the 2 Affymetrix probes non-associated to functional known genes (Figure 10).

Twenty-four out of these 48 genes showed connection by direct and/or indirect protein-protein interaction and were functionally linked in biological processes such as immune system processes, lymphocyte activation and differentiation, regulation of apoptosis, response to stress and growth factor and signal transduction (Figure 11A). Moreover, from the main molecular interaction network, one main densely connected sub-network clearly emerged, encompassing 6 genes (*RELB, NFKB2, BIRC3, TNFAIP3, NFKBIA* and *CYLD*) (Figure 11B). The biological processes implicated with these genes are related to the regulation of the NF-κB pathway, apoptosis and the adaptive immune response. One interesting gene is the deubiquitinating enzyme CYLD described as interacting with Tax-1 and implicated in the regulation of the signaling function of Tax-1 [58]. CYLD plays an important role in the regulation of pathways leading to NF-κB activation and contributes to the regulation of cell survival, proliferation and differentiation [59]. Moreover, CYLD is also able to inhibit HDAC6, a member of the HDAC family whose major substrate is α-tubulin, has become a target for drug development to treat cancer due to its major contribution in oncogenic cell transformation [60]. Interestingly, it was recently shown that using an HDAC inhibitor combined with an inhibitor of the reverse transcriptase caused a strong decrease in the proviral load of STLV-1 infected monkeys [61]. Although all Tax proteins induced overexpression of CYLD, the levels of expression in Tax-1 and Tax-3 are higher compared to Tax-2 (8.3, 5.3 and 8.6 fold for Tax-1, -2 and -3 respectively) (Figure 10). Therefore, understanding the mechanism of alteration of this gene’s expression following Tax transduction could be of interest.

Altogether, these results suggest that the 48 genes that are up-regulated in Tax1, -2 and -3 transduced cells are likely to be essential during HTLV infection, when Tax is expressed.

### The Tax-3-induced Expression Profile is More Similar to that of Tax-1 than Tax-2

Interestingly, while performing comparative analysis of the molecular profile associated with Tax-1, -2 or -3 expression in MOLT4 cells, we noticed that among 118 genes that were common between Tax-1 and Tax-3 expressing cells, 70 were exclusively deregulated by Tax-1 and Tax-3 (Figure 9). By comparison, 49 and 56 genes are commonly expressed in Tax-1/Tax-2 or Tax-2/Tax-3 expressing cells. Among them only 1 and 8 genes were exclusively deregulated between Tax-1/Tax-2 and between Tax-2/Tax-3 respectively (Figure 9).

It therefore appears that Tax-3 expression induced a set of genes, which are also deregulated in Tax-1 expressing cells. Among this list of genes, only 26 were already described in the HTLV-1 literature (noted with an asterisk): *ALCAM, BHLHE40, CDH1, CFLAR, GADD45B, GATA3, HLA-C, ICAM1, IL10RA, IL15, INPP1, ITPR1, JUNB, MEF2C, NEDD9, NEDD4L, NR4A1, PLAT, PTP4A3, REL, SORD, SSTR2, TANK, TNFSF9, TRAF1* and *TRIB1*), whereas 44 were not associated with HTLV infection and will deserve further studies (Figure 12). A significant fraction of those genes is implicated in cell growth (*ARHGAP31, GADD45B, IL15, TNFSF9, ZFP36L1*); cell differentiation (*BHLHE40, RUNX2*); regulation of proliferation (*BTG2, LHX2*); regulation of apoptosis (*CFLAR, CSRNP1, RASSF2, RASSF4, RBMS3*), T-cell development (*GATA3*); modulation of NF-κB signaling pathway (*TANK, TIFA, TNIP1, TRAF1*) but also oncogenesis (*JUNB, PTP4A3, REL* and *YAP1*). Furthermore, most of these 70 genes were poorly expressed in Tax-2 transduced MOLT4 cells (Figure 12). As an example, the growth arrest and DNA-damage-inducible beta GADD45B gene and the Inter-Cellular Adhesion Molecule 1 ICAM-1/CD54 gene, are highly expressed in both Tax-1 and Tax-3 transduced cells but expressed at similar levels in control cells and Tax-2 transduced cells (Figure 12). Consistent with the Affymetrix data, we confirmed a lower level of ICAM1 protein expression in Tax-2 transduced cells compared to Tax-1 and Tax-3 transduced cells (Figure 13). Consistent with our findings, it was previously shown that Tax-1 and Tax-2 have a differential transactivation activity on the ICAM1 promoter. In T-cell lines (Jurkat, MOLT4 and CEM) and non T-cell lines (HeLa), Tax-1 can strongly activate the ICAM1 promoter whereas Tax-2 can only activate this promoter in HeLa cells [62]. We also performed western blot analyses on RAB9A, another gene up-regulated in Tax-1 and Tax-3 transduced cells. RAB9A belongs to the Ras oncogene superfamily. Consistent with the Affymetrix data, RAB9A protein level was increased in Tax-1 and Tax-3 expressing cells compared to Tax-2 expressing cells (Figure 13). Of note, this protein has been recently implicated in the trafficking of HIV-1 from the late endosome to the Trans-Golgi network during the formation of the bridging conduit or “tunneling nanotubes” (TNT) in macrophages [63]. It is therefore possible, at least in HTLV-1 infected cells, that RAB9A, in cooperation with the auxiliary protein p8, could be implicated in the formation of cellular conduits (nanotube like structures) as recently described [64].

Molecular interactions existing between the 70 genes composing the molecular signature specific to Tax-1 and Tax-3 were further examined as a biological network (Figure 14). The majority of the genes are functionally linked in biological processes characteristic of Tax-1 expressing HTLV-1 infected T-cells: regulation of transcription and apoptosis, activation of the NF-κB cascade, T-cell mediated immunity and induction of cell proliferation and differentiation.

Together with the results presented in Figure 6, these data suggest that the gene expression profile of Tax-3 is more closely related to that of Tax-1 than Tax-2 and that Tax-3 activates a number of genes implicated in cellular processes and mechanisms possibly leading to the cellular transformation.

## Discussion

Using DNA microarray analysis, we compared for the first time the gene expression profile of CD4^+^ T-cells or 293 T cells expressing the Tax proteins from HTLV-1, -2 and -3 viruses. Microarray technology allowed us to examine the profile for over 47,000 transcripts and in conjunction with bioinformatic tools, we were able to group the deregulated genes into functionally associated pathways. To date, the majority of gene profiling was done on HTLV-1 infected cell lines or ATLL patient samples, focusing on differences in transcription profiles of disease/transformed state versus control T-cells [40], [41], [42], [43], [44], [45], [46], [47], [48], [49]. Our study was aimed at delineating Tax regulated genes induced upon expression of the viral protein in CD4^+^ T-cells or 293 T cells and further to distinguish gene profiles among the three Tax proteins and therefore, was finally aimed at determining the oncogenic potential of Tax-3.

HTLV-1, HTLV-2 and HTLV-3 Tax proteins are required for viral replication and function as transactivators of proviral transcription. Tax-1 and Tax-2 have also been shown to play a pivotal role in the immortalization and transformation of infected cells by altering cellular gene expression and protein function [20], [26], [34], [36], [65]. While some differences between Tax-1 and Tax-2 activity on cellular transcription factors such as p53, NF-κB and co-activator binding [26], [33], [66], [67], [68], little has been done to compare Tax-3 to other Tax proteins [16], [17]. Because of the lack of data regarding the comparison of the three HTLV viruses, we first focused our functional analysis on genes that were commonly up-regulated by all Tax proteins. In MOLT4 cells, 48 of the 239 Tax-1 up-regulated genes were similarly increased by both Tax-2 and Tax-3 proteins. Among these genes, seventeen were also up-regulated in 293 T cells (Figure S2A). Not surprisingly, the majority of the genes identified were involved in cellular growth processes. These genes encompass oncogenes (*BCL3, MAP3K8, RELB*), anti-apoptotic genes (*BIRC3, IER3*), *CD83* gene, tumor suppressor genes (*EGR1*), signal transduction genes (*GEM, JAK3, NFKB2, NFKBIA, NFBIZ, PTGER4, TNFAIP3*), innate immunity genes (*IFIT3*) and cell adhesion genes (*JAM2, VCAM1*) Figure S2B). Consistent with previous reports, thirteen of these genes were shown to be up-regulated following HTLV-1 infection (or Tax-1 expression) [40], [41], [42], [43], [44], [45], [46], [47], [48], [49]. Our study also uncovered four genes (*IER3, NFKBIZ, PTGER4* and *JAM2*) (non identified genes were also presented in the Table S2). Among them, IER3 plays an important role in the cell cycle control and apoptosis (Figure S2B) but also in inflammation and tumorigenesis by interfering with certain signaling pathways, in particular NF-κB, MAPK/ERK and PI3K/Akt [69]. Our results therefore suggest that during HTLV infection, all three Tax proteins function in a similar manner to activate a subset of cellular genes involved in cell cycle progression.

We also assessed the gene expression profiles of Tax-3 transduced cells. Our results demonstrate that Tax-3 expression activates a significant number of genes that were previously shown to be characteristic of HTLV-1. These genes are implicated in the regulation of transcription and apoptosis, T-cell activation and differentiation, and leukocyte migration. Remarkably, although Tax proteins share considerable structural and functional similarity, our comparison of Tax-3 with the other two Tax proteins demonstrated that a discrete expression profile for each Tax protein was evident. Interestingly, of the 189 genes up-regulated by Tax-3, 70 genes were similarly increased by expression of the oncogenic protein Tax-1. In contrast, only one was uniquely common between Tax-1 and Tax-2 and only 8 between Tax-2 and Tax-3. Interestingly a similar pattern was observed in 293 T cells, where 33 genes were common between Tax-1 and Tax-3 whereas only 7 and 17 are commonly up-regulated by Tax-1 and Tax-2 and Tax-2 and Tax-3, respectively (Figure S1). Similarly, in T- and non T- cell types, Tax-3/Tax-1 down-regulated genes were more frequent than genes down-regulated by Tax-1/Tax-2 or Tax-2/Tax-3 (Figure S3).

These results strongly suggest that Tax-3 and Tax-1 are related proteins in their transcriptional activity and are clearly distinct from Tax-2. This is consistent with one previous molecular study which suggested that HTLV-1 and HTLV-3 Tax proteins were functionally related: (i) at the amino acid levels with the presence of a PBM in Tax-1 and Tax-3 but not in Tax-2. This domain plays a key role in HTLV-1-induced cell proliferation *in vitro* and facilitates viral persistence *in vivo* [34], [36], [37]. We can assume that the presence of the PBM in Tax-3 sequence increases its transforming potential like Tax-1; (ii) with a strong nuclear localization for Tax-1 and Tax-3 but not for Tax-2; and (iii) the ability of Tax-1 and Tax-3 to transrepress some cellular gene products like p53 or c-Myb [16].

Eighteen of these genes have already been associated with HTLV-1 infection, while the remaining are described for the first time here as HTLV-targeted genes and will require further studies. Among those, the following are of particular interest in the context of HTLV infection: the activated leukocyte cell adhesion molecule (ALCAM), which plays a role in the binding of T- and B-cells to activated leukocytes, as well as in interactions between cells of the nervous system; the Rho GTPase activating protein 31 (*ARHGAP31*) gene which encodes a GTPase-activating protein (GAP) for RAC1 and CDC42 (CDC42 was shown to be an essential regulator of the cell polarity and important in MTOC/Tax polarization in HTLV infected T cells [51]); the gene encoding a microtubule-associated protein 7 (MAP7) that may play an important role during reorganization of microtubules during polarization and differentiation of epithelial cells.

PDLIM2 suppresses HTLV-1 Tax-mediated tumorigenesis by targeting Tax to the nuclear matrix for proteasomal degradation [70]. PDLIM4, which is up-regulated by Tax-1 and Tax-3, has a tumor suppression function and is repressed by DNA methylation in many cancers [71]. Several genes of the Ras oncogene family are also up-regulated by both proteins: RAB27A, RAB9A, RASFF2, RASSF4. For example, RAB27A may be involved in protein transport and small GTPase mediated signal transduction. These few examples of Tax-1/Tax-3-up-regulated genes demonstrate possible new implications of these Tax proteins in cellular processes that have not been described until now. Hence, further studies on the genes up-regulated by Tax-3 and Tax-1 are necessary for improving knowledge and new mechanism on Tax-3/HTLV-3 infection.

Tax is not the only HTLV-1 protein that is important for cell transformation [72]. Indeed, it has been shown that HBZ, together with Tax-1, participate in cell proliferation *in vivo* and *in vitro* [20] and more recently in cell transformation [73]. Interestingly, like HBZ, the antisense protein of HTLV-2, APH-2, is expressed *in vivo*, is able to interact with CREB and repress Tax-2-mediated transcription but does not promote cell proliferation [21], [22]. However, APH-2, like APH-3 and -4, doesn’t possess a classical bZIP domain and demonstrates a differential subcellular localization compared to HBZ [74]. This highlights other phenotypical differences among HTLV viruses.

In conclusion, we have performed a high throughput analysis to study the global gene expression profiles of T- and non T-cells expressing Tax-1, Tax-2 or Tax-3. Independently of the cell type, we identified a set of genes whose expression is commonly affected by all Tax proteins and that are hence characteristic of the HTLV infection. We have also shown that Tax-3 and Tax-1 are closely related in terms of induced cellular transcriptional profiles. The majority of Tax-1/Tax-3 up-regulated genes are functionally linked in biological processes characteristic of Tax-expressing HTLV-1-infected T-cells: regulation of transcription and apoptosis, activation of the NF-κB cascade, T-cell mediated immunity and induction of cell proliferation and differentiation. Given the prime role of Tax and of its transcriptional activities in the initiation of the HTLV-1 induced ATLL, it is possible that differences in Tax properties might account for differences in physiopathological outcomes among HTLVs. HTLV-3 might share pathogenic features with HTLV-1 *in vivo* and that the apparent lack of symptoms in HTLV-3-infected individuals might only be a result of the very limited number of individuals studied so far.

## Materials and Methods

### Cells Culture

293 T and MOLT4 cells were cultured in DMEM-GLUTAMAX-I and RPMI-GLUTAMAX-I (Gibco, Invitrogen), respectively, complemented with 10% fetal bovine serum (FBS) (Gibco, Invitrogen), and antibiotics (penicillin-streptomycin, PAA). Cell lines were maintained at 37°C in 5% CO2. 293 T **(**CRL-11268™) and MOLT4 **(**CRL-1582™) cells were obtained from ATCC.

### Lentiviral Particles Production

Ten centimeters plates were pre-coated with poly-L-lysine (Sigma) for 30 min before plating 6.10^6^ of 293 T cells. The following day, cells were transfected with 4.68 µg of psPAX-2 (Gag/Pol, Addgene), 2.52 µg of pMD2.G (Env, Addgene) and 9 µg of pSDM101-FLAG-TAX (LENTI-Flag-Tax), or pSDM101-empty (LENTI-IRES-GFP) (Dasgupta unpublished data) using LipoD293 reagent (Gentaur). Tax-1, Tax-2B, and Tax-3 cDNA were amplified from pSG5M-Tax-1, pSG5M-Tax-2, and pSG5M-Tax-3, respectively [16], [29]. Seventy-two hours post-transfection, supernatants were collected, centrifuged for 5 min at 3000 rpm then filtered through a 22 µm filter (Millipore). Stock of lentiviral particles were conserved at −80°C.

### Transduction

293 T: 4.10^6^ cells were plated on 10 cm dishes. Twenty-four hours later, lentiviral particles (necessary for obtaining 100% transduced cells) were incubated with 8 µg/ml of polybrene (Sigma) then added on the cells in 4 ml of DMEM-stable L-Glutamine with antibiotics, without FBS. Six hours post-transduction, medium was replaced with complete medium.

MOLT4∶5.10^6^ cells were prepared in 5 ml of DMEM-stable L-Glutamine with antibiotics without FBS. Lentiviral particles were incubated with 8 µg/ml of polybrene (Sigma) and added to the cells. Cells were then centrifuged for 1 hour at 3000 rpm, at room temperature. Cell pellets were gently resuspended and complete medium was added 6 hours post-transduction.

### Microarray Experiments

Seventy-two hours post-transduction, total RNAs were isolated from 293 T or MOLT4 cells using RNA-bee reagent (Tel-Test, Inc). One hundred micrograms of RNA were purified using the RNeasy mini columns kit (Qiagen). cRNA probes were prepared following Affymetrix instructions and hybridized to Human Genome-U133-plus 2.0 GeneChip® oligonucleotide arrays (Affymetrix). Normalization of raw data and clustering analysis were performed using GeneChip® Operating software (MAS5.0) and BRB Array Tools software (NIH, Rockville).

### RT-PCR

Seventy-two hours post-transduction, total RNAs from 293 T-transduced cells were purified on silica columns using the RNeasy mini kit (Qiagen). To avoid DNA carryover, samples were treated twice with the DNase I RNase-free DNAs set (Qiagen). Five hundred nanograms of total RNAs were then used as a matrix for RT-PCR using the one step RT-PCR kit from Qiagen. PCRs were performed following manufacturer’s instructions with primers designed using Affymetrix sequence probes (Table S1). Annealing temperatures were 52°C for Tax-1, Tax-2, GADD45B, BIRC-3 and ICAM1 primers; and 59°C for Tax-3 and GAPDH primers.

### Western Blot

Seventy-two hours post-transduction, cells were collected and washed with PBS. Proteins were extracted (50 mM Tris-HCl pH 8, 120 mM NaCl, 5 mM EDTA, 0.5% NP40, 1 mM PMSF, 1 mM DTT, 50 mM NaF, 0.2 mM Na_3_VO_4_) in the presence of protease inhibitors (Complete-EDTA-free, Roche) then quantified using the Bradford reagent assay (Biorad). Samples were loaded into 4–12% NU-PAGE gels (Invitrogen). Following electrophoresis, proteins were transferred onto a PVDF membrane using the I-blot system (Invitrogen). Membranes were blocked in a 5% milk/PBS-Tween 0,05% solution for 1 h, then incubated overnight with primary antibody (anti-Flag clone M2 (Sigma), anti-β-actin clone AC74 (Sigma), anti-BIRC3/cIAP2 clone E40 (Abcam), anti-RAB9A sc53145 (Santa Cruz Biotechnology) or anti-ICAM1 ab66100 (Abcam). The next day, the membrane was washed and incubated either with anti-rabbit or with anti-mouse horseradish peroxidase-conjugated secondary antibodies (GE Healthcare). The membranes were then developed using the SuperSignal West Pico (Pierce/Thermoscientific) or ECL plus kit (GE Healthcare).

### Immunofluorescence

293 T cells were transduced with Lenti-Flag-Tax lentiviruses. Seventy two hours later, cells were fixed in 4% PFA solution (Sigma) for 20 min then permeabilized with 0.5% Triton X-100 (Sigma) for 10 min. Following washes with PBS, cells were preincubated with a 5% PBS/milk solution then incubated with anti-Flag M2 antibody (Sigma) at a 1∶100 dilution in 5% PBS/milk for 1 h at 37°C. Samples were then stained with CY3-conjugated goat anti-IgG mouse (Amersham Biosciences) at a 1/1000 dilution in 5% PBS/milk for 1 h at 37°C. Nucleic acids were stained with DAPI-containing mounting medium (Vectashield, Vector Laboratories) and cells were visualized with a Zeiss Axioplan 2 imaging microscope, X40, and the the SimplePCI software (Hamamatsu).

### Luciferase Assays

Forty-eight hours post-tranduction, 293 T cells were transiently transfected with HTLV-1-LTR-luc (250 ng) or NF-κB-luc (500 ng), using Polyfect reagent (Qiagen). All the transfections were carried out in the presence of a phRG-TK vector (10 ng) in order to normalize the results for transfection efficiency. Reporter activities were assayed 24 h post-transfection using the dual-luciferase reporter assay system (Promega). Luciferase assays were performed with the Veritas microplate luminometer (Promega).

### Functional Analysis of Microarray Data

Identification of set of genes specifically or commonly deregulated in Tax-1, Tax-2 or Tax-3-transduced cells (293 T or MOLT4) *versus* control (Lenti-IRES-GFP)-transduced cells was performed by Venn diagram analysis. Gene ontology (GO) enrichment was evaluated by DAVID Bioinformatics web-tool (Database for Annotation, Visualization and Integrated Discovery) (http://david.abcc.ncifcrf.gov/) [75], [76], [77], [78], [79]. The DAVID Functional Annotation Tool application provided functional cluster of genes, according to similar and redundant GO terms. Only GO Biological Process terms were used for this analysis.

Biological networks were built and visualized into Cytoscape software [80], [81], [82] according to the following analysis workflow:

- Interactions among members of a selected set of genes are retrieved with STRING (Search Tool for the Retrieval of Interacting Genes/Proteins) (http://string-db.org/) [83], [84], [85]. STRING is a database of known and predicted protein interaction, including direct (physical) and indirect (functional) association, deriving from different sources: genomic context, co-expression, high-throughput experiment (BIND, DIP, GRID, HPRD, IntAct, MINT, DIP), curated data of various databases (BioCarta, BioCyc, GO, KEGG, Reactome) and text-mining (PubMed).
- Each interaction retrieved from one given source in STRING is given a score, which reflects the accuracy of the interaction. Combination of the scores obtained from different sources allows calculation of a combined score [83], [84]. On the graphics, width of edges reflects the value of this combined score, and hence, the global accuracy of the interaction (ranging from 0.4 to 1).
- Circles (genes) are colorized according their associated GO terms (Biological Process) using BINGO [86] and GOlorize [87] plugins implemented into Cytoscape.
- The whole network is analyzed using the MCODE plugin (Molecular Complex detection) [85], which detects densely-connected regions of molecular interaction networks solely based on connectivity data. These subnetworks are re-analyzed according to their GO enrichment with BINGO and GOlorize.

## Supporting Information

Figure S1 **Functional analysis of cellular genes up-regulated following Tax expression in 293 T cells.** Venn diagram representation performed on 318 cellular genes up-regulated by Tax expression in 293 T cells (cut-off: 3-fold over the control).
(TIF)

Figure S2 **Functional analysis of cellular genes commonly deregulated following Tax expression in 293 T and MOLT4 cells.** (A) Representation of the 17 cellular genes deregulated both in 293 T and MOLT4 cells during Lenti-Flag-Tax-1, -2 and -3 transduction using Heat Map analysis (log transformation and mean centered data performed in Cluster and TreeView softwares. The mean of fold change expression was indicated on the right of each graphic. *Genes were already reported in HTLV literature. (B) Schematic representation of the 17 cellular genes implicated in molecular interactions, using the STRING software. Width of the lines reflects the score of molecular interaction and the circles are colored according to the GO Biological Process association. The color legend is indicated in the table below the network. Each color represents the main GO terms associated with genes composing the network, identified by BINGO analysis (Hypergeometric test and Benjamini & Hochberg False Discovery Rate (FDR) correction; significance level <0.05).
(TIF)

Figure S3 **Functional analysis of cellular genes down-regulated following Tax expression in MOLT4 and 293 T cells.** Venn diagram representation performed on cellular genes down-regulated by Tax expression in (A) MOLT4 and (B) 293 T cells (cut-off: 3-fold over the control).
(TIF)

Table S1 **List of primers designed for RT-PCR experiments.**
(XLS)

Table S2 **List of genes up-regulated following Tax expression in 293 T and MOLT4 transduced cells.**
(XLS)

Table S3 **List of genes down-regulated following Tax expression in 293 T and MOLT4 transduced cells.**
(XLS)
