# Supplementary figures and images for: The Transcription Profile of Tax-3 Is More Similar to Tax-1 than Tax-2: Insights into HTLV-3 Potential Leukemogenic Properties
Source: PLoS One. 2012 Jul 20;7(7):e41003. doi: 10.1371/journal.pone.0041003 (PMC3401231; doi:10.1371/journal.pone.0041003)

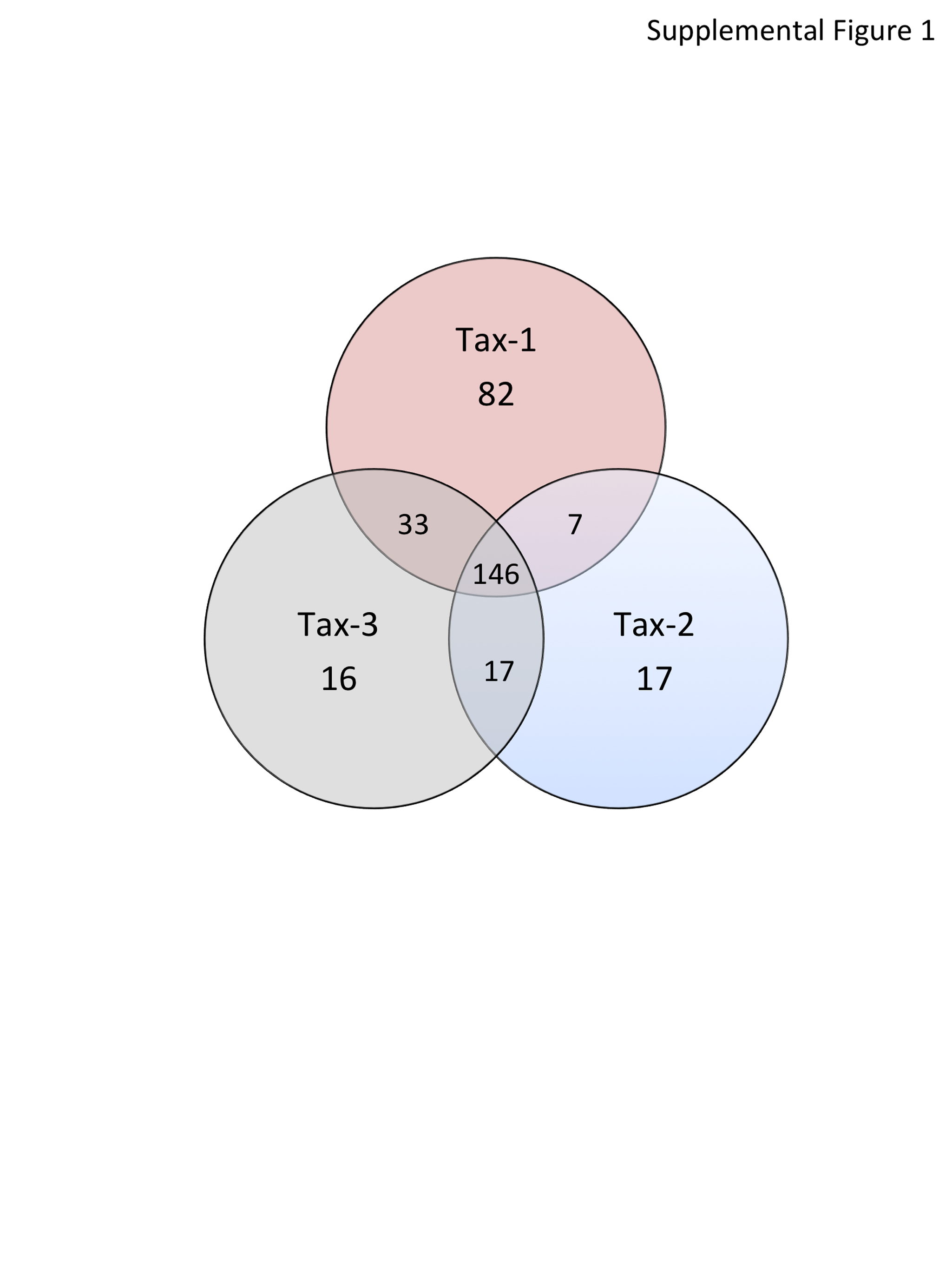

Supplement: Figure S1 — Functional analysis of cellular genes up-regulated following Tax expression in 293 T cells. Venn diagram representation performed on 318 cellular genes up-regulated by Tax expression in 293 T cells (cut-off: 3-fold over the control). (TIF) [file pone.0041003.s001.tif]

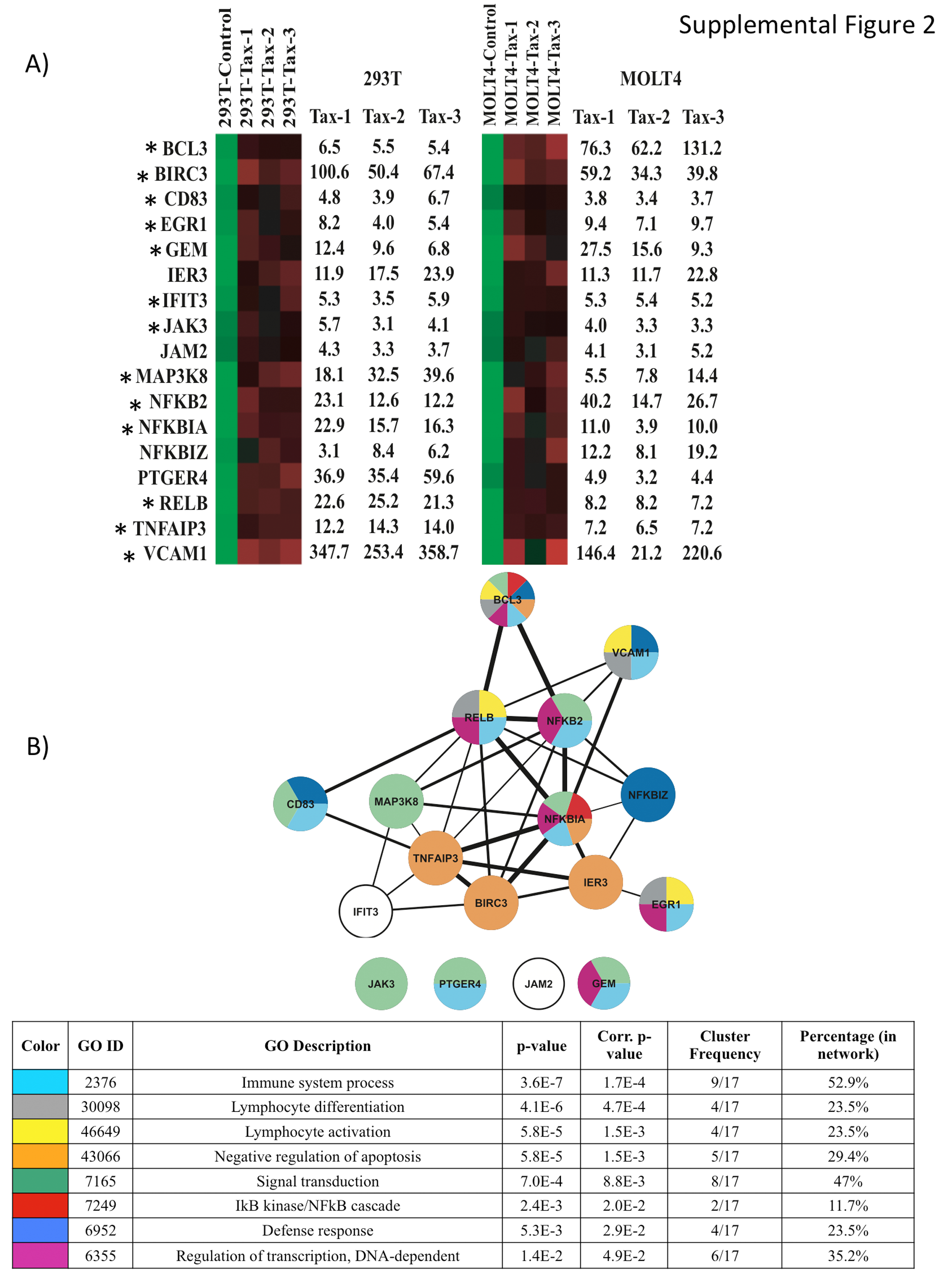

Supplement: Figure S2 — Functional analysis of cellular genes commonly deregulated following Tax expression in 293 T and MOLT4 cells. (A) Representation of the 17 cellular genes deregulated both in 293 T and MOLT4 cells during Lenti-Flag-Tax-1, -2 and -3 transduction using Heat Map analysis (log transformation and mean centered data performed in Cluster and TreeView softwares. The mean of fold change expression was indicated on the right of each graphic. *Genes were already reported in HTLV literature. (B) Schematic representation of the 17 cellular genes implicated in molecular interactions, using the STRING software. Width of the lines reflects the score of molecular interaction and the circles are colored according to the GO Biological Process association. The color legend is indicated in the table below the network. Each color represents the main GO terms associated with genes composing the network, identified by BINGO analysis (Hypergeometric test and Benjamini & Hochberg False Discovery Rate (FDR) correction; significance level <0.05). (TIF) [file pone.0041003.s002.tif]

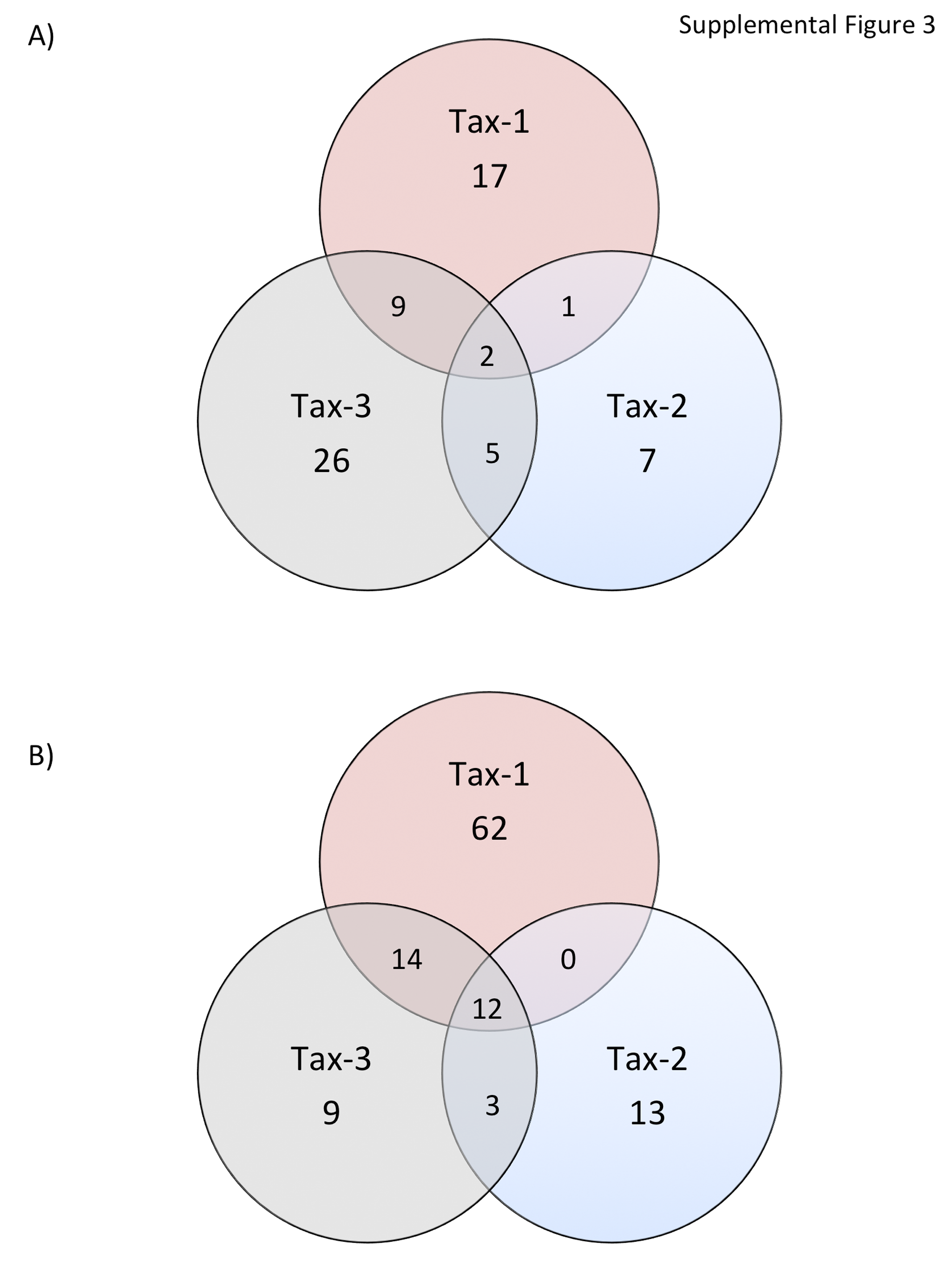

Supplement: Figure S3 — Functional analysis of cellular genes down-regulated following Tax expression in MOLT4 and 293 T cells. Venn diagram representation performed on cellular genes down-regulated by Tax expression in (A) MOLT4 and (B) 293 T cells (cut-off: 3-fold over the control). (TIF) [file pone.0041003.s003.tif]
